# How Much Is Too Much? Assessment of Prey Consumption by Magellanic Penguins in Patagonian Colonies

**DOI:** 10.1371/journal.pone.0051487

**Published:** 2012-12-12

**Authors:** Juan E. Sala, Rory P. Wilson, Flavio Quintana

**Affiliations:** 1 Centro Nacional Patagónico, Consejo Nacional de Investigaciones Científicas y Técnicas (CONICET), Puerto Madryn, Chubut, Argentina; 2 Swansea Moving Animal Research Team, Biosciences, College of Science, Swansea University, Swansea, Wales, United Kingdom; 3 Wildlife Conservation Society, Ciudad Autónoma de Buenos Aires, Argentina; Institut Pluridisciplinaire Hubert Curien, France

## Abstract

Penguins are major consumers in the southern oceans although quantification of this has been problematic. One suggestion proposes the use of points of inflection in diving profiles (‘wiggles’) for this, a method that has been validated for the estimation of prey consumption by Magellanic penguins (*Spheniscus magellanicus*) by Simeone and Wilson (2003). Following them, we used wiggles from 31 depth logger-equipped Magellanic penguins foraging from four Patagonian colonies; Punta Norte (PN), Bahía Bustamente (BB), Puerto Deseado (PD) and Puerto San Julián (PSJ), all located in Argentina between 42–49° S, to estimate the prey captured and calculate the catch per unit time (CPUT) for birds foraging during the early chick-rearing period. Numbers of prey caught and CPUT were significantly different between colonies. Birds from PD caught the highest number of prey per foraging trip, with CPUT values of 68±19 prey per hour underwater (almost two times greater than for the three remaining colonies). We modeled consumption from these data and calculate that the world Magellanic penguin population consumes about 2 million tons of prey per year. Possible errors in this calculation are discussed. Despite this, the analysis of wiggles seems a powerful and simple tool to begin to quantify prey consumption by Magellanic penguins, allowing comparison between different breeding sites. The total number of wiggles and/or CPUT do not reflect, by themselves, the availability of food for each colony, as the number of prey consumed by foraging trip is strongly associated with the energy content and wet mass of each colony-specific ‘prey type’. Individuals consuming more profitable prey could be optimizing the time spent underwater, thereby optimizing the energy expenditure associated with the dives.

## Introduction

Birds are major consumers in the marine environment, with estimations of their consumption amounting to between 55.6 and 83.7 million tonnes per year [1] and, as such, are assumed to play an important role in modulating marine food web structure [e.g. [2]–[6]]. This highlights the critical need for determination of precisely how much birds consume even though our methods for doing this are rather crude; while prey types can be elucidated using stomach contents, guano and/or pellet analysis [e.g. [7],[8],[9]], determination of actual rates of prey consumption by seabirds is not trivial. In fact, in a general sense, our understanding of this is slowly being built up via a suite of widely disparate methodologies. Small cameras have been used to document direct evidence of feeding habits [e.g. [10],[11]] and attempts have been made to determine food intake by, for instance, examining change in stomach temperature [e.g. [12],[13]], changes in stomach pH [14], or by documenting particular behaviours that animals use at sea in order to secure prey [15], [16]. Probably the most promising approach uses animal-attached logging systems for determining ingestion rates based on high frequency recording of parameters specifically associated with prey ingestion. The best examples are beak-opening angles [16] and oesophageal temperature [3], [17] because seabirds cannot ingest prey without opening their beaks and incurring an oesophageal temperature drop although small prey may not always be registered by oesophageal drops (see [17]).

Simeone and Wilson [18] and Bost et al. [19] used these systems in free-living penguins to propose a simple and apparently effective method for estimating prey consumption. They noted that prey capture was almost invariably associated with a consistent pattern in the temporal variation of depth data recorded by high-frequency recording time-depth loggers [cf. [20],[21],[22]] because most penguin species apparently catch their prey by lunging at them from the underneath [3], [11], [21]–[25]. This observation, which has since been proposed to be valid for 4 species of penguin: *Spheniscus magellanicus*, *Aptenodytes forsteri*, *Aptenodytes patagonicus* and *Pygoscelis adeliae* [e.g. [17],[18],[19],[22],[25]]; has allowed researchers to re-interpret time-depth data derived from loggers deployed on penguins without having to resort to the complexities and difficulties associated with the use of beak or oesophageal sensors [17], although its use would appear less rigorous for species that take small prey items (see [19] for a discussion of this).

We use data stemming from variation in depth associated with prey capture published by Simeone and Wilson [18] for Magellanic penguins (*Spheniscus magellanicus*) to derive rates of food consumption for this species operating from four colonies of the Patagonian coast of Argentina. In order to do this, we make a number of assumptions and approximations in a procedure that is a first best guess of this important metric. Penguins are, in general, considered important in structuring marine food webs of the Southern Hemisphere Oceans because they account for about 90% of the avian biomass [26], but Magellanic penguins, in particular, are ranked 20^th^ in terms of projected global annual food consumption of all seabird species [1], and are thus expected to have a substantial effect on the trophic functioning of associated marine ecosystems [1]. We calculate rates of prey ingestion and derive ‘catch per unit time’ (CPUT) indices for birds from all four sites and then use information on colony-specific diet [27], [28], [29] and its energetic values [30] to determine the rate of energy acquisition as a function of locality. This approach allows us to construct a first estimate of predator impact on the ecosystems and may help explain penguin population trends over recent years.

## Materials and Methods

### Study Sites and Period

The Magellanic penguin breeds in colonies distributed along the coast of Argentina from about 41° S to almost 55° S latitude [31]. We conducted fieldwork during early chick-rearing, between November and December 2005, 2006, 2007 and 2008, at four colonies along the Patagonian coast (aprox. 1150 km coastline), Argentina: Punta Norte/San Lorenzo (42° 04′ S, 63° 49′ W), Bahía Bustamante (45° 10′ S, 66° 29′ W), Puerto Deseado (47° 45′ S, 65° 52′ W) and Puerto San Julián (49° 16′ S, 67° 42′ W). All necessary permits for the described field studies were obtained from Subsecretaria de Turismo y Áreas Protegidas and Dirección de Fauna y Flora Silvestre (Chubut Province, Argentina), and Dirección de Fauna Provincial, Consejo Agrario Provincial (Santa Cruz Province, Argentina).

### Deployment of Devices

A total of 82 Magellanic penguins brooding small chicks was equipped with one of two different types of recording technology (see below). Birds were carefully removed from their nests using a clipboard [32] and then equipped with devices which were attached to the feathers of their lower backs using overlapping strips of waterproof tape [33] to minimize hydrodynamic drag [34]. Every effort was taken to minimize the stress caused to the birds during manipulation and the procedure was completed in less than five minutes, after which the birds were immediately returned to their nests. All devices were retrieved after a single foraging trip, being recovered the moment birds returned from the sea. Thus, no single individual contributed more data to the set than any other. All birds equipped with devices continued to breed normally during the study period.

#### Daily Diaries

Thirty-five birds equipped with multichannel Daily Diaries (DD) archival tags (see [35] for details) which recorded data with 22 bit resolution at rates of 6 to 9 Hz in 13 channels. Recording channels relevant for the present study were triaxial body acceleration (range = –4 to 4 *g*) (see [35] and references therein) and pressure (0.5 to 20 bar). Accuracy on all channels was better than 1% of full-scale deflection except for depth, where accuracy was better than 0.01%. The devices were made to be streamlined and had maximum dimensions of 70×40×10 mm (L×W×H), constituting 3.8% of the penguin cross-sectional area. They weighed 68 g, which is less than 1.5% of the mean weight of an adult Magellanic penguin (mean: 4.4 kg; range: 2.7–7.2 kg; [36]).

#### GPS-TDlogs

Forty-seven Magellanic penguins were also equipped with GPS loggers (GPS-TDlog, Earth and Ocean Technologies, Kiel, Germany) which recorded depth, latitude and longitude. The horizontal accuracy of the positional fixes (recorded at 1 Hz when the penguins were not underwater) was better than 5 m for 90% of fixes (GPS-TDlog Manual). The depth data was recorded at 0.5 Hz and was accurate to 0.3 m. Data were stored in a 2-Mbyte flash memory. Loggers had a hydrodynamic, waterproof housing measuring 96×39×27 mm (L×W×H), comprising ∼ 6.5% of the cross-sectional area of the bird, and a total mass of 75 g, which is *ca*. 1.7% of the mean Magellanic penguin body mass.

### Data Analysis of Diving Behaviour

Penguin diving behaviour was analysed using bespoke software (SNOOP; Gareth Thomas, Free Software, Swansea, Wales, United Kingdom), specially designed to detect automatically the three characteristic phases of a dive (descent, bottom and ascent phase), based on changes in the rate of descent/ascent [cf. [22]] and analyze the times and depths associated with each one of them. We considered “dives” to be all submersions that exceeded 1.5 metres depth and defined bottom phases, during which the penguins are most likely to hunt [37] and catch most of their prey [18], [38], according to three conditions; they could only occur (i) at depths >85% of the maximum depth recorded during the dive, (ii) they were delimited by two points of inflection in the rate of change of depth (following the descent phase and preceding the ascent), and (iii) when the overall rate of change of depth for the whole period did not exceed 0.25 m s^−1^ [22].

### Classification of the Foraging Trip Segments and Time Activity Budget

Penguin foraging trips were divided into three distinctive segments; outbound, foraging area, and inbound. Birds leaving the colony were considered to be undertaking the outbound section of the trip until the moment the first dive exceeded a depth of 10 m after which the birds were considered to be foraging [25]. Foraging behaviour could be further confirmed using acceleration and depth data from the DD because variation in the depth profile took the form of undulations [18], [20], [21] accompanied by increases in flipper beat frequencies associated with prey chases shown by the heave acceleration [21]. The end of the foraging phase and the start of the return phase was also clear, being defined by regular, shallow (<10 m) dives with a clear parabolic shape [36]. All parameters studied correspond to the foraging segment of trip (see above). Using the definitions above, we calculated the total number of dives per foraging phase, the time spent underwater during foraging, the maximum dive depths reached per foraging dive and the rate of foraging dives, defined by the number of foraging dives divided by the number of hours foraging during the foraging phase of the trip.

### Estimation of Prey Consumption and Catch Per Unit Time (CPUT)

Simeone and Wilson [18] report that undulations - also termed ‘wiggles’ [cf. [39]] - in the dive profile (presented graphically as depth against time) indicate when Magellanic penguins catch prey. They define a wiggle as a change in depth greater than 0.3 m over 1 s and note that there are three possible scenarios in the analysis of wiggles: (i) a wiggle occurs that does not result in the consumption of a prey (type A), (ii) consumption occurs without registering a wiggle (type B), and (iii) a wiggle corresponds to the consumption of prey (type C). In the latter case, the authors also analyzed the probability of penguins could have caught more than one prey for every wiggle. Simeone and Wilson [18] proposed that the ‘total number of capture events’ (TCE) could be represented by the following formula:
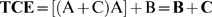
(1)using their above definition of wiggles and concluded that the best estimate of prey consumption is, in fact, to consider that each detected wiggle represents of the consumption of a single prey because the errors cancel each other out. Simeone and Wilson [18] also note that depth sampling for this approach should not be less than 0.5 Hz (the lowest recording interval used - in our GPS-TDlogs). This conclusion was subsequently reinforced by the work of Bost et al. [19], Hanuise et al. [17] and Wilson et al. [21]. Importantly, although most penguins (7 individuals) in the study by Simeone and Wilson [18] came from Cabo Virgenes, a colony we did not study, three birds came from Punta Norte and Puerto San Julián, to which can be added a further four individuals studied by Wilson et al. [21] foraging from Punta Norte, Puerto San Julián and Bahía Bustamante, all our study colonies, which showed the same patterns with respect to wiggles and prey capture.

We identified wiggles according to the criteria set-out by Simeone and Wilson [18] for the high temporal resolution Daily Diary data but sub-sampled these data to simulate the lower sampling regime of the GPS-TDlogs to ascertain that a wiggle could also be defined as a change of depth of >2 m over a 4 s interval or >1 m over a 2 s interval (which, all other things being equal, equates to >0.5 m over a 1 s interval), something that accords closely with the value of >0.3 m over a 1 s interval presented in both Simeone and Wilson [18] and Wilson et al. [21]. In short, either 2 or 3 serial points of inflection (SPI) adhering to the vertical velocity rules within the appropriate time frame (see above) were defined as a single wiggle (cf. [22]). In order to assess the extent of potential differences between devices in their capacity to provide data allowing the detection of wiggles, we analysed derived results according to colony and device (see statistics below). We used the number of wiggles divided by the total time spent underwater during foraging as a measure of ‘catch per unit time’ (CPUT), only using birds where complete foraging trips were recorded (Table 1). We note that wiggles are generally considered to be indicative of prey pursuit in penguins [e.g. [17],[20],[21],[22],[24],[25],[40]] but that the precise validity of this assumption is critical to our assessment of prey consumption and ‘catch per unit time’. Against this, Simeone and Wilson’s [18] study to assess the validity of wiggles was conducted rigorously on the Magellanic penguin, our study species here.

**Table 1:** Site of deployment and type of device fitted to Magellanic penguins from Patagonian colonies during the early-chick rearing period between November and December 2005 to 2008.

Site	Study Year	Type of device	# of birds with data	# of birds with complete trips	# of dives
Punta Norte	2008	GPS-TDlog	9	9	6448
		Daily Diary	5	1	2508
Bahía Bustamante	2005	Daily Diary	3	3	1483
	2006	GPS-TDlog	1	1	641
		Daily Diary	6	2*	2993
	2007	Daily Diary	1	1	512
Puerto Deseado	2006	Daily Diary	6	4	5245
Puerto San Julián	2007	GPS-TDlog	6	6	6126
		Daily Diary	7	6*	8994
**Total**			**44**	**33**	**34950**

* For statistical analysis we removed two individuals (one of each colony; see text) as they were considered outliers (they had values that deviated 2.5 times from the standard deviation of the average for the colony to which they belong).

### Determination of a Standard Colony-specific “Prey Type”

To determine the most appropriate prey type for each of our studied colonies, we used data published in the scientific literature on the percentage contribution to diet (by number) of species consumed by Magellanic penguins from three of the four colonies studied [27], [28], [29]. Since there are no diet studies published pertaining to Bahía Bustamante (45° 10′ S, 66° 29′ W), we assumed that birds from this site had a percentage composition of prey equal to that at Cabo Dos Bahías (44° 54′ S, 65° 32′ W), the closest colony (∼ 80 km) where dietary information is available [27]. Recent data of diet composition of breeding penguins from Bahía Bustamente (D. Gonzalez-Zevallos and P. Yorio, unpublished data), taken by stomach flushing [41], accord with our assumption. The average wet mass of each prey type consumed was extracted from Scolaro et al. [28], where, according to the authors, values are fairly constant among colonies, even over the range of prey species taken by the birds. We note, however, that annual variation in prey type, size and energy content may change our derivations accordingly. Values for energy density (ED), expressed as kJ g^−1^ of wet mass, for each prey type, were taken from Ciancio et al. [30]. Thus, for example, birds from Puerto Deseado consume essentially Sprat (*Sprattus fuegensis*), Squid (*Loligo gahi*), Silverside (*Odontesthes smitti*) and Hake (*Merluccius hubbsi*) [27] which have mean wet masses of 13.1, 11.5, 2.5 and 46.8 grams per individual prey-item, respectively [28], and corresponding energy densities of 7.15, 4.95, 4.57 and 4.08 kJ g^−1^, respectively [30] (Table 2). Thus, the mean energies provided by each individual sprat, squid and silverside are 13.1×7.15 = 93.7 kJ, 11.5×4.95 = 56.9 kJ, 2.5×4.57 = 11.4 kJ, and 46.8×4.08 = 190.9 kJ, respectively. Since, the three different prey types consumed at this site constitute 15, 30, 54, and 1% of the prey caught, by number, for sprat, squid, silverside and hake, respectively, the average energy value for a ‘mean’ Puerto Deseado prey, would be (93.7×0.15)+(56.9×0.30)+(11.4×0.54)+(190.9×0.01) = 39.2 kJ. This process was applied to all colonies to derive standard colony-specific prey types defined by their energy value, and we followed the same logic to get the total wet mass of each standard prey type (Table 2).

**Table 2:** Derivation of a standard colony-specific “Prey Type” based on the relative contributions of various species in the diet of Magellanic penguins from the four studied colonies in Patagonia, Argentina. The energetic value of a single standard “Prey Type” is composed of an amalgamation of all the species caught by penguins at each locality (see text).

Colony	Punta Norte		Bahía Bustamante		Puerto Deseado		Puerto San Julián	
Prey Item (ED)*	%†	Wet MassContribution (g)‡	%†	Wet MassContribution (g)‡	%†	Wet MassContribution (g)‡	%†	Wet MassContribution (g)‡
Anchovy (5.5 kJ g^−1^)	98	19.3	54	10.6	0	0	0	0
Sprat (7.15 kJ g^−1^)	0	0	0	0	15	2	64	8.4
Cephalopods (4.95 kJ g^−1^)	0.5	0.1	1	0.1	30	3.5	8	0.9
Hake (4.08 kJ g^−1^)	0.8	0.4	45	21.1	1	0.5	0	0
Silverside (4.57 kJ g^−1^)	0.7	0.02	0	0	54	1.4	28	0.7
**“Prey Type”**	**EC (kJ)**	**Total Wet Mass (g)**	**EC (kJ)**	**Total Wet Mass (g)**	**EC (kJ)**	**Total Wet Mass (g)**	**EC (kJ)**	**Total Wet Mass (g)**
	**109.1**	**19.8**	**145.5**	**31.8**	**39.2**	**7.4**	**67.7**	**10.0**

* The Energy Density (ED) values, expressed as kJ per gram of wet mass, were extracted from Ciancio et al. [30] for Anchovy (*Engraulis anchoita*); Sprat (*Sprattus fuegensis*); Squid (*Loligo gahi*) (as an example of Cephalopods); Hake (*Merluccius hubbsi*); and Silverside (*Odontesthes smitti*) [27], [28].

† Importance of prey species (%) by number for Magellanic penguins consumed for each colony were extracted from Frere at al. [27], Scolaro et al. [28] and Wilson et al. [29].

‡ The average weight of each prey, and with which we calculate the percentage contribution of wet mass in each case, was extracted from Scolaro et al. [28]. The Energy Content (EC, kJ) of the “Prey Type” of each colony was calculated as the energy density (ED) muliplied by the wet mass of each prey, according to their relative contribution, and then adding the partial contributions. Total Wet Mass (g) represents the sum of partial contributions of wet mass of each prey, as well as the wet weight of each “Prey Type”.

By multiplying the total number of wiggles recorded for each penguin by the energy content of the colony-specific ‘prey type’ (Table 2), we attained a mean value of the total energy consumed per foraging trip by colony. The same procedure was followed to calculate the average total wet mass consumed per foraging trip on each location.

### Statistical Analysis

For all parameters studied we obtained a value for every individual where we had fully documented foraging trips (n = 33) before deriving a grand mean per colony (see Table 1). Where a significant difference was detected using ANOVA, differences between means were tested with the Student-Newman-Keuls *post hoc* test [[42], cf. [43]]. Where necessary, we log-transformed the data in order to satisfy the ANOVA assumptions of normality and homocedacy [42]. Proportional data were averaged for individual penguins and arcsin-transformed to normalize them [42]. To evaluate possible differences in the detectability of wiggles due to the different devices used (i.e. using different sampling frequencies), and thus validate our classification methodology of wiggles (see above), we compared the number of wiggles per dive (for each colony separately) using general linear mixed-effects models (GLMMs; i.e. to account for repeated measures, and avoid pseudo-replication), with restricted maximum likelihood estimations (REML), and where the identity of the bird was considered as a random factor and the ‘device’ as a fixed factor [44]. To deal with non-Gaussian distributions, we used GLMMs with poisson errors and log link function corrected for overdispersion [44]. Thus, to compare the effect of any difference of detectability of wiggles because of the different recording frequencies, we compared the model considering the ‘device’ as a fixed effect vs. the model that did not consider it (i.e. only considering the random effect of different individuals), using a chi-square test. The premise that precedes all this is that the higher the recording frequency (i.e. as in Daily Diaries compared to GPS-TDlogs), the greater the number of wiggles per dive recorded. This analysis was performed for the three colonies that had data from both types of devices, namely Punta Norte, Bahía Bustamante and Puerto San Julián (see Table 1). For all statistical tests, the threshold was taken to be 5%. Data are given as mean ± SD unless otherwise noted.

## Results

The wiggle classification adopted for the data obtained from the two different device types (see ‘Materials and Methods’) showed that the number of wiggles per dive during the foraging phase of trips did not differ, at any site (where both device types were used; see Table 1), between the different recording systems used, and their associated recording frequencies (GLMMs; Punta Norte: *Χ^2^* = 0.52, P = 0.47, N_(dives)_ = 4025, N_(ID)_ = 10; Bahía Bustamante: *Χ^2^* = 0.13, P = 0.72, N_(dives)_ = 1934, N_(ID)_ = 6; Puerto San Julián: *Χ^2^* = 1.40, P = 0.24, N_(dives)_ = 8034, N_(ID)_ = 11). This strongly implies that the capability to detect a wiggle in dive was the same for the two sampling frequencies used in this work.

Of the 82 devices deployed reliable data were only obtained from 44 units (see Table 1) (there were 38 cases of e.g. battery exhaustion or sensor failure before trips ended etc.). Specifically however, complete trip depth records were available from 33 Magellanic penguins providing more than 1014 hours of time at-sea. During this time we analyzed a total of 34,950 dives made by animals carrying instruments (Table 1). However, for statistical analysis we removed two individuals (one bird from Bahía Bustamante spent less than an hour ostensibly foraging, at a mean dive depth of 8.3 m (compared to a colony mean of 52 m) while another bird, from Puerto San Julián, apparently spent a total of 73.5 h foraging (compared to a colony mean of 17.0 h) (Table 3). The maximum number of wiggles per dive was 6 (1.21±0.62 dive^−1^). The maximum dive depth recorded was 85.5 m (19.5±16.9 m) and the maximum dive duration was 188 s (62.2±36.4 s).

**Table 3:** Foraging parameters for Magellanic penguins with fully documented foraging trips (n = 31) during the early chick-rearing period, according to colony. Average values are given (SD), along with range [Max-Min]. Mean values and significant statistical test are showed in bold.

Colony (n)	Punta Norte (10)	Bahía Bustamante (6)	Puerto Deseado (4)	Puerto San Julián (11)	F_(df = 30)_	P
Duration of the foragingphase (h)*	**11.6** (3.0) [16.5–7.7]^d^	**12.5** (4.8) [19.7–6.0]	**16.7** (8.4) [28.1–8.0]	**17.0** (4.1) [25.2–12.9]^d^	**3.1**	**0.0431**
N^o^ of foraging dives	**402.4** (135.6) [647–235]^d^	**322.3** (100.2) [402–136]a,c	**629.8** (306.3) [1074–372]^a^	**730.7** (360.8) [1683–360]c,d	**6.8**	**0.0015**
Diving rate (foragingdives h^−1^)*	**34.4** (5.9) [43.0–27.8]	**26.3** (4.7) [30.9–19.1]^c^	**41.4** (19.0) [68.1–22.9]	**42.3** (13.8) [70.5–27.3]^c^	**3.6**	**0.0262**
Time underwater (h)	**8.0** (1.8) [10.7–5.6]	**7.9** (2.5) [10.4–4.0]	**12.6** (6.0) [20.6–6.0]	**10.7** (3.0) [15.8–7.3]	**3.2**	**0.0399**
Percentage timediving (%)*	**69.5** (4.3) [76.1–62.5]^d^	**64.2** (7.6) [72.7–52.5]^a^	**75.9** (3.1) [80.2–73.2]a,b	**62.8** (6.9) [69.3–47.3]b,d	**5.9**	**0.003**
Total wiggles	**294.7** (105.7) [457–178]^e^	**265.7** (100.3) [353–124]^a^	**895.1** (555.9) [1602–320]a,b,e	**431.0** (174.1) [718–212]^b^	**6.7**	**0.0017**
Wiggles per dive	**0.74** (0.13) [0.94–0.55]^e^	**0.82** (0.15) [0.94–0.54]^a^	**1.43** (0.60) [1.98–0.59]a,b,e	**0.61** (0.13) [0.81–0.38]^b^	**8.6**	**0.0004**
CPUT (wiggles h^−1^)†	**36.1** (6.4) [44.3–24.5]^e^	**33.1** (3.9) [37.4–26.5]^a^	**67.9** (19.2) [90.1–50.1]a,b,e	**40.0** (11.5) [65.9–27.6]^b^	**9.2**	**0.0002**
Wet mass consumedper dive (g)	**14.6** (2.6) [18.5–10.9]d,e,f	**26.2** (4.6) [29.8–17.1]a,c,f	**10.6** (4.4) [14.6–4.3]a,b,e	**6.1** (1.3) [8.1–3.8]b,c,d	**43.1**	**<0.0001**
Energy consumedper dive (kJ)	**80.4** (14.1) [102.1–60.1]d,e,f	**119.7** (21.2) [136.5–78.1]a,c,f	**56.0** (23.4) [77.5–23.0]a,e	**41.4** (9.1) [54.9–25.8]c,d	**35.5**	**<0.0001**
Total wet massconsumed (kg)	**5.8** (2.1) [9.0–3.5]	**8.5** (3.2) [11.2–4.0]^c^	**6.6** (4.1) [11.9–2.4]	**4.3** (1.7) [7.2–2.1]^c^	**3.6**	**0.0256**
Total energyconsumed (MJ)	**32.2** (11.5) [49.8–19.4]	**38.7** (14.6) [51.3–18.1]	**35.1** (21.8) [62.8–12.6]	**29.2** (11.8) [48.6–14.4]	0.67	0.5809

One-way ANOVA was used to compare between colonies, with Student-Newman-Keuls (S-N-K) post-test. The significant differences (P<0.05) in the results of post-hoc S-N-K’s contrast are shown by the superscript letters as follow:

a Bahía Bustamante vs. Puerto Deseado;

b Puerto Deseado vs. Puerto San Julián;

c Bahía Bustamante vs. Puerto San Julián;

d Puerto San Julián vs. Punta Norte;

e Puerto Deseado vs. Punta Norte; and,

f Bahía Bustamante vs. Punta Norte.

* Calculated using a corrected time at sea value, subtracting the hours of darkness from the total time at sea. †Number of wiggles per hour underwater.

### Dive Behaviour

At least one penguin from each colony spent a night at sea during their foraging trips (overnight trips). During the night, the penguins stayed on average 6.3±1.0 hours without apparent feeding behaviour; diving activity was minimal and no dive exceeded a depth of 10 metres. The breeding site with the highest proportion of overnight trips was Puerto San Julián, where nearly 30% of equipped animals spent the night at sea. For Puerto Deseado, this proportion was 25%, for Bahía Bustamante it was 14% while it was lowest in the Punta Norte colony at 10%.

The average time spent in the foraging phase per trip was slightly different between sites, with birds from Puerto San Julián spending more time than those from Punta Norte (F_(1,30)_ = 3.11, P = 0.04; Table 3). There were large differences between colonies in the number of dives made by the penguins during foraging (F_(1,30)_ = 6.77, P<0.002; Table 3), with Bahía Bustamante and Puerto San Julián showing the lowest and highest number of foraging dives per trip, respectively (Table 3). There were slight inter-colony differences in the number of foraging dives made per hour (diving rate) (F_(1,30) = _3.60, P = 0.03; Table 3), and in the time spent underwater during foraging (F_(1,30) = _3.18, P = 0.04; Table 3), with, in both cases, Bahía Bustamante and Punta Norte being the sites where the lowest values were recorded, Puerto San Julián and Puerto Deseado having the highest (Table 3). The percentage of time diving during the foraging phase (in relation to recovery time at surface) was significantly different among colonies (F_(1,30) = _5.93, P = 0.003), with the highest percentage in Puerto Deseado (75.9±3.1%) and the lowest in Puerto San Julián (62.8±6.9%) (Table 3).

**Figure 1:**
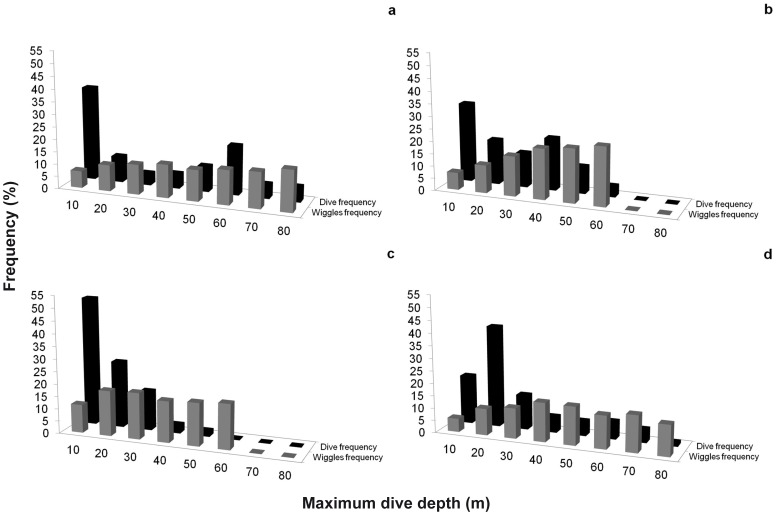
Frequency distribution (%) of dives and wiggles as a function of maximum dive depth (10-m intervals) for the four studied colonies. Bahía Bustamante (a), Puerto Deseado (b), Puerto San Julián (c) and Punta Norte (d).

There appeared to be marked inter-colony difference in the frequency distribution of dive depths (using depth intervals of 10 m) (Fig. 1). The high incidence of travelling dives (generally in the range 1.5 to 10 m; see [29]) resulted in a substantial left-hand skew in depth frequency distribution of dives for almost all colonies (with the possible exception of Punta Norte; Fig. 1). These travelling dives accounted for 52% of all dives of the Puerto San Julián birds while for the remaining colonies they accounted for about half this value (range: 20 to 38%, Fig. 1). The frequency distribution of dives for the other depth intervals (11 to 80 m) showed a mode in the 51–60 m interval for Bahía Bustamante (Fig. 1a) and one in the 41–50 m interval for Puerto Deseado (Fig. 1b). In contrast, birds from Puerto San Julián and Punta Norte executed 94 and 74%, respectively, of their foraging dives to within the first ∼ 30 m of the water column (Fig. 1c and d).

There were substantial differences between colonies in the mean number of wiggles per foraging trip (F_(1,30) = _6.70, P = 0.002; Table 3), with Puerto Deseado having more than three times the number of wiggles of Bahía Bustamante or Punta Norte, and twice the number of wiggles recorded in Puerto San Julián (Table 3). The average number of wiggles per foraging dive also differed widely among colonies (F_(1,30)_ = 8.56, P = 0.0004), with the mean value registered in Puerto Deseado being almost the double that those from Punta Norte or Bahía Bustamante, and being nearly 2.5 times higher than that found in Puerto San Julián (Table 3). Puerto Deseado had the highest CPUT, being almost twice that of the remaining colonies (F_(1,30) = _9.16, P = 0.002; Table 3).

The depth-dependent frequency distributions of wiggles appeared to differ between colonies, although, in general, the first 10 metres of seawater showed few wiggles (typically between 5 and 11% of the total wiggles) (Fig. 1). This apart, penguins from all colonies either showed increasing wiggles with increasing depth (R^2^ = 0.89, F_(1,7)_ = 20.28, P = 0.004 and R^2^ = 0.99, F_(1,5)_ = 265.70, P = 0.0004, for Bahía Bustamante and Puerto Deseado, respectively) (Fig. 1a and b) or had a number of wiggles that increased with depth before reaching a plateau (e.g. R^2^ = 0.91, F_(1,6)_ = 24.88, P = 0.003, for Punta Norte; Fig. 1d; while for Puerto San Julián the quadratic relationship was not significant; R^2^ = 0.59, F_(1,5)_ = 2.20, P = 0.26; Fig. 1c).

### Estimates of Food Consumption Rates

We found large differences in the estimated mean wet mass and energy consumed by the penguins per foraging dive (F_(1,30)_ = 43.11, P<0.0001 and F_(1,30)_ = 35.52, P<0.0001, respectively) (Table 3). Penguins from Bahía Bustamante apparently acquired the greatest wet mass, and energy, per foraging dive while birds from Puerto San Julián had the lowest (Table 3). Penguins from Punta Norte and Puerto Deseado showed intermediate values (Table 3). There was, however, a notable inverse relationship between the mass ingested per dive and the mean number of foraging dives conducted per trip for the different colonies (Table 3). At the level of the foraging trip, there were marked differences in the average amount of wet mass consumed per trip among colonies (F_(1,30)_ = 3.62, P = 0.0256; Table 3). For example, birds from Bahía Bustamante ingested the double the quantities of birds from Puerto San Julián (Table 3). Despite all differences between colonies described above, we found no difference in the total energy consumed by the penguins per foraging trip (F_(1,30)_ = 0.67, P = 0.58).

### Correlates of Prey Mass and Energy

**Figure 2:**
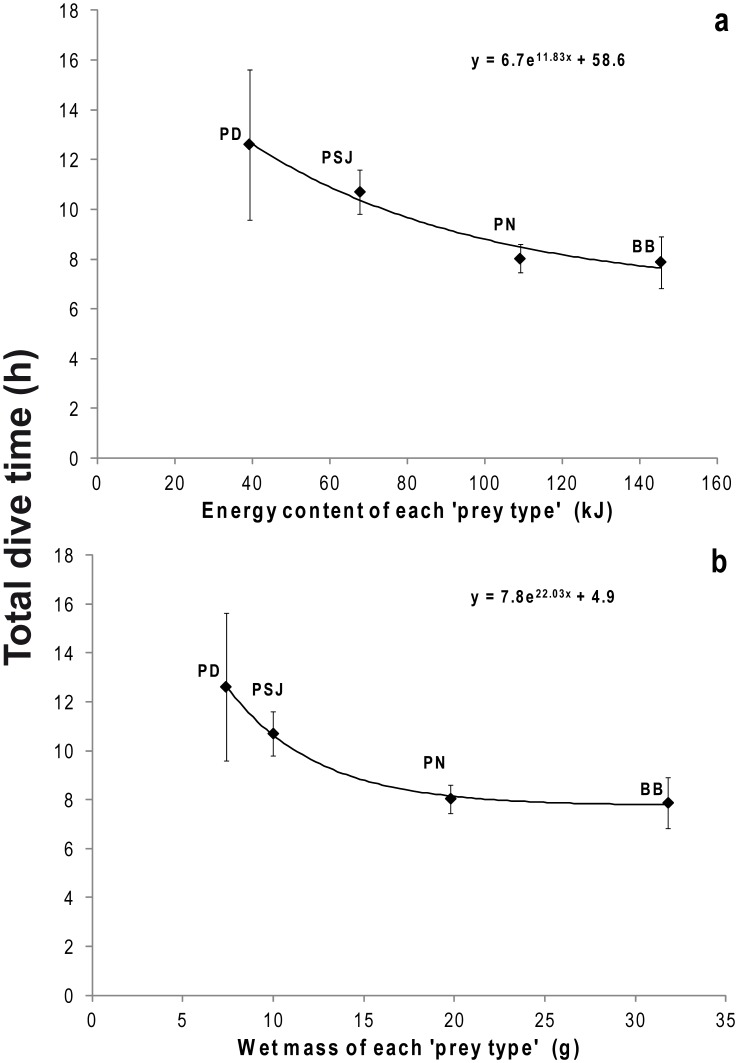
Energy content and wet mass of each colony-specific ‘prey type’ related with total dive time (h). Relationship between the energy content (kJ) and wet mass (g) and the average total dive time (h) (**a** and **b**, respectively). Bahía Bustamante (BB), Puerto Deseado (PD), Puerto San Julián (PSJ) and Punta Norte (PN).

**Figure 3:**
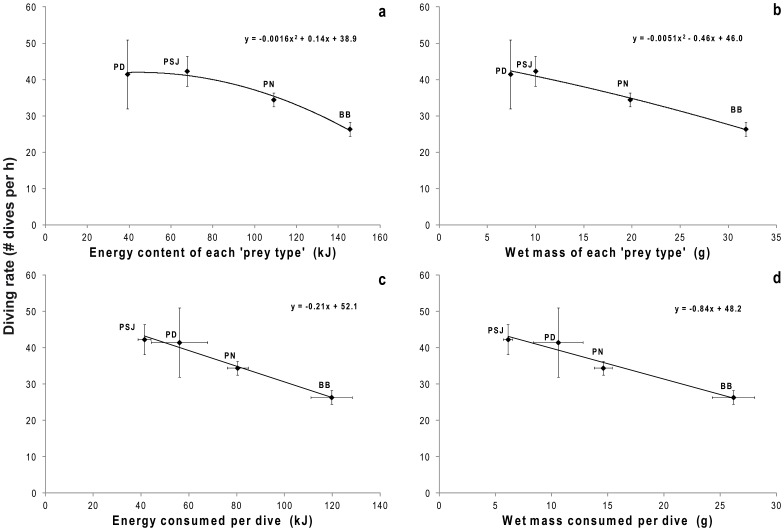
Diving rate in relation to the content and consumption of energy and wet mass per colony according with ‘prey type’. Relationship between the average diving rate (# dives h^−1^) per colony and: **a**) energy content of each ‘prey type’ (kJ), **b**) wet mass of each ‘prey type’ (g), **c**) energy consumed per dive (kJ), and **d**) wet mass consumed per dive (g). Bahía Bustamante (BB), Puerto Deseado (PD), Puerto San Julián (PSJ) and Punta Norte (PN).

The time spent diving of penguins on each colony was negatively correlated to the energy content and wet mass of each colony-specific ‘prey type’ (R^2^ = 0.98, F_(1,4)_ = 40.1, P = 0.02 and R^2^ = 0.99, F_(1,4)_ = 607.7, P = 0.0016; respectively; Fig. 2a and b). Additionally, colony-specific dive rate was negatively correlated with both energy content and the wet mass of each colony-specific ‘prey type’ (R^2^ = 0.98, F_(1,4)_ = 59.7, P = 0.016 and R^2^ = 0.98, F_(1,4)_ = 55.2, P = 0.018; respectively; Fig. 3a and b) and mean colony-specific dive rate was lower in those colonies where birds acquired more energy (R^2^ = 0.98, F_(1,4)_ = 110.7, P = 0.009; Fig. 3c) and greater mass of prey per dive (R^2^ = 0.95, F_(1,4)_ = 50.0, P = 0.023; Fig. 3d).

## Discussion

### General Foraging Patterns and Inter-colony Comparisons

Some authors have shown that Magellanic penguins from different colonies adjust their behaviour at-sea to accord with local conditions [e.g. [29]] and prey type [18], [20], [29]. Our data on foraging behaviour showed this too. We identified, for example, significant differences in the number of foraging dives, time underwater, diving rate and number of wiggles displayed by penguins from different colonies, all these factors presumably being due to the different colony-specific prey species. But the matter may be more complex than penguin behaviour simply being a reflection of prey behaviour. Absolute prey abundance presumably also plays a role. Breeding penguins from the two northern-most colonies (Punta Norte and Bahía Bustamante) showed the lowest diving rates and spent the least time underwater during the foraging phase of their trips, in relation to the other colonies. This may be due to them having the most profitable prey acquisition, both in terms of wet mass and energy per dive (Table 3). This constrasts with the percentage time diving being highest in birds from the Puerto Deseado colony (Table 3), where penguins were eating smaller prey items with lower values of wet mass and/or energy content. Generally, we would expect unfavorable scenarios to be defined by birds spending increased time underwater (prey can only be encountered during swimming underwater), with individuals using this strategy to increase the probability of encountering prey per unit time spent at sea (cf. [45] and references therein). Based on this, penguins from Puerto Deseado appear to be working harder than birds from other colonies, which might explain why the population at this site seems to be faring badly [46]. However, foraging effort and return should be also examined within the context of the whole foraging trip, rather than just the foraging phase, because time and energy is invested in commuting between the nesting and foraging sites. Ostensibly low rates of prey capture in the foraging area are easier to defend if commuting time is minimized and *vice versa*. Sala et al. [46] examine the total times allocated to commuting by penguins from the four colonies studied here and report these to be (means of) 10.9, 15.9, 10.4 and 11.9 h for Punta Norte, Bahía Bustamante, Puerto San Julián and Puerto Deseado, respectively, showing general similarity except for Bahía Bustamante, where commuting times are about a third longer. If the total time spent in the foraging area is added to that spent commuting (using data from Sala et al. [46]) and this then converted into a rate of energy acquisition for the whole foraging trip (by reference to data acquired in this study – Table 3), the four study colonies of Punta Norte, Bahía Bustamante, Puerto San Julián and Puerto Deseado have energy acquisition rates of 366, 277, 364 and 386 J s^−1^, respectively, showing, again, remarkable similarity overall except for birds from Bahía Bustamante, which are about 26% lower. The case of this colony shows that high rates of energy consumed per dive during the foraging period (Table 3) do not always compensate for foraging areas situated far from the breeding site and may help explain, again, why the penguin population at Bahía Bustamante is also faring badly [46].

Overall though, it would seem that a large part of apparent inter-colony variability in, for example, prey size, prey energy content (Table 2), prey encounter rate (Table 3), distance of foraging site from the colony [46] and depth of prey (Fig. 1), etc., can be compensated by penguins varying the rate at which they work via, for example, the percentage of time they spend underwater (Table 3) and the speed at which they travel [46]. Indeed, these compensations can be seen in figures 2 and 3, and in Table 3. For example, a lower amount of energy obtained on each dive, is offset by an increase in the dive frequency (Table 3) and similar compensations are apparent for catch per unit of time (CPUT) and energy consumed by each dive (Table 3) as well as for the mean total number of wiggles and the energy content of each colony-specific prey type (Table 3).

Penguins also apparently compensate the maximum depth of dives with prey abundance because, for example, birds from Punta Norte and Bahía Bustamante performed deeper dives than penguins from Puerto San Julián and Puerto Deseado but also show higher numbers of wiggles (and therefore the estimated prey consumption) with increasing maximum diving depth, reason enough to justify the strategy [cf. [22]].

### Expected Versus Observed Consumption Rates

A standard method to estimate consumption by any animal is to derive it from field metabolic rate (FMR) [e.g. [1],[47],[48]] or by summing the energy expenditures from known time/activity budgets assuming that animals balance energy lost with energy gained [29], [49], [50]. The tags deployed in this study allow us to approximate this second approach here if we make a few basic assumptions about Magellanic penguin activity-specific metabolic rate. Here, we assume that the time spent at sea is roughly divided into that swimming underwater and that resting at the sea surface [36] and that the metabolic rate for birds at sea overall is about 6.6 × standard metabolic rate (SMR) (this value taken from the congeneric African penguin (*Spheniscus demersus*) – [47]). Lasiewski and Dawson’s [51] general equation for non-passerine birds gives an SMR of 11 W for a typical 4 kg Magellanic penguin [cf. [47]] so that at-sea costs based on foraging trip durations of 24.5, 38.7, 41.4 and 27 h for Punta Norte, Bahía Bustamante, Puerto Deseado and Puerto San Julián, respectively (using data from Sala et al. [46]), would be 6.4, 10.2, 10.8 and 7.1 MJ, respectively. If birds adhere very approximately to a two-day forage-brooding cycle rhythm [cf. [36],[47]] and have an on-land metabolic rate of 1.7 × SMR [47], which amounts to 18.7 W, birds from Punta Norte, Bahía Bustamante, Puerto Deseado and Puerto San Julián would have minimum foraging-brooding cycle costs of 8.0, 10.8, 11.2 and 8.5 MJ, respectively. The respective “prey type” energy densities are 5.51, 4.58, 5.30 and 6.77 kJ g^−1^ (see Table 2), which, given an assimilation efficiency of 77% [52], [53], translates into metabolizable energy contents of 4.24, 3.52, 4.08 and 5.21 kJ g^−1^, respectively. Thus, to cover their energetic costs of a two day foraging-brooding cycle, birds from Punta Norte, Bahía Bustamante, Puerto Deseado and Puerto San Julián would need to consume about 1.9, 3.0, 2.7 and 1.6 kg of food per foraging trip, respectively. These figures are substantially higher than those estimated for the African penguin by Nagy et al. [47] using doubly labelled water but accord in as much as they are derived using the Nagy et al. [47] estimates. As such, the increased values can be traced directly back to the larger mass of the Magellanic penguin and the fact that they spend much longer periods at sea than the African penguins in Nagy et al.’s [47] study. They are, however, far less than the amounts than the 5.83, 8.45, 6.62 and 4.31 kg that the Magellanic penguins were calculated to consume (Table 3) and the several kilogram difference for all colonies more than makes up for the small amounts that the penguins might be feeding their small chicks (the *ca.* 3 kg African penguin feeding small chicks brings back a mean of 150 g in the stomach for the brood per foraging trip; see [54]).

How is this discrepancy to be explained? Our estimates of prey consumption will depend critically on the premise that a single undulation or ‘wiggle’ in the depth data over time, as defined by Simeone and Wilson [18], genuinely represents the capture of a single prey, and that this premise is generally valid for all prey types. Although the general concept that wiggles are indicative of prey capture has been adopted by the penguins researcher community [20], [21], [22], [24], [25], [40], two groups of authors, Bost et al. [19] and Hanuise et al. [17] have explicitly tested it (in King and Adélie penguins) and report good concurrence. In support of this, using ‘Daily Diary’ tags, from which the 3-dimensional trajectory of swimming animals can be reconstructed with sub-second resolution [35], Wilson et al. [21] have described the mechanism by which the undulation occurs in the Magellanic penguin. They showed that the positive buoyancy of foraging penguins allows them to accelerate towards the water surface without work by the flippers, catching fish from the underneath where they are most visible to predators ([3] and references therein). This rapid action therefore facilitates the capture of highly mobile school fish, which constitute their major prey ([36] and references therein), with minimum use of energy. An inevitable consequence of the manoeuvre is that it elicits an abrupt rise in the water column, something that manifests itself as a ‘wiggle’ in the depth data. Thus, as stated by Simeone and Wilson [18], it seems reasonable to expect wiggles to be diagnostic of prey capture in general, with the proviso that some prey items, particularly those that are atypical, may be missed using this metric. If, on the contrary, the amount consumed mirrors that needed and no more (see above), penguins would be, on average, catching one prey item every 3.1, 2.8, 2.45 and 2.7 wiggles, for birds from Punta Norte, Bahía Bustamante, Puerto Deseado and Puerto San Julián, respectively (these figures obtained by dividing the total number of wiggles recorded (Table 3) by the mass ingested using the Nagy *et al*.-based model, itself divided by the mean mass of prey items; Table 2). These figures are about 200% higher than those found to be the case by Simeone and Wilson [18] and are difficult to consolidate with the prey attack strategy of the Magellanic penguin as detailed by Wilson et al. [21].

A number of recent lines of evidence also suggest that the norm for determining seabird consumption figures, using known assimilation efficiencies and derived, or measured, energy expenditure, assuming that birds consume only as much as they need [e.g. [1],[47],[55]] may not be correct. In the only studies where prey consumption has been measured ‘directly’, i.e. via stomach temperature sensors [37] or beak angle sensors [16], [20], masses of food consumed have routinely greatly exceeded that necessary for maintenance. For example, a recent study by Humphries et al. [56] using stomach temperature sensors, determined that Wandering albatrosses (*Diomedea exulans*) consumed four times the amount they needed, while similar work on King penguins by Pütz and Bost [57] found that these birds could, exceptionally, consume over double their body mass in food per day. Finally, Wilson et al. [5] used mandibular sensors to determine that Magellanic penguins from Cabo Virgenes, Argentina, ingested up to 60% of their body mass over eight hours foraging. All this, in addition to the results apparent from our study, suggest that it might be germane to consider that some seabirds may be able to consume much higher quantities than we have previously supposed. We attempt to do this below by using a model which, although subject to a number of assumptions, at least gives us a framework with which to examine the matter.

**Figure 4:**
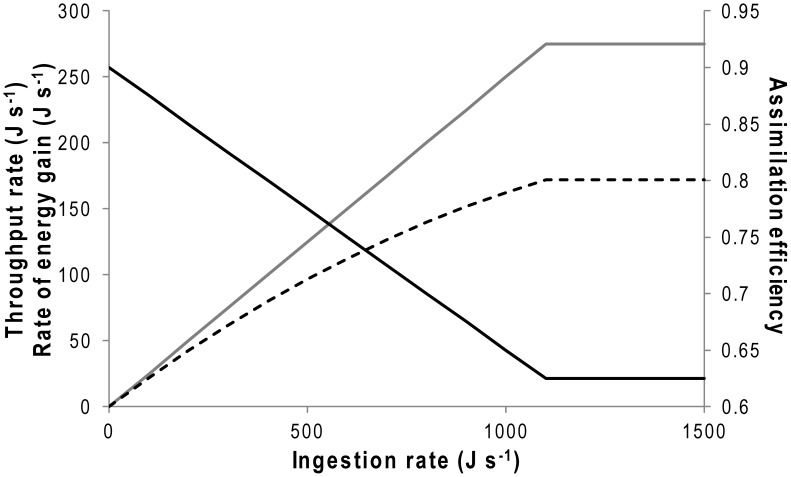
Model of penguin digestion. Parameters used in a simplified model of penguin digestion, which assumes that, once the stomach is full, the throughput rate (grey line) increases linearly with consumption rate (here taken to be = 0.25 X) until a maximum (225 J s^−1^). The model also assumes that the percentage of the energy in the ingesta that is absorbed is modulated by the assimilation efficiency, which decreases linearly with throughput rate (starting at 0.9 ( = 90%) at an ingestion rate of *ca.* 0 J s^−1^, down by 0.1 for every 400 J s^−1^ ingested to a minimum of 0.625 (at ingestion rates of 1100 J s^−1^); black line). A consequence of these is that the rate of energy gain follows an approximately log-type function against consumption rate (dashed line).

**Figure 5:**
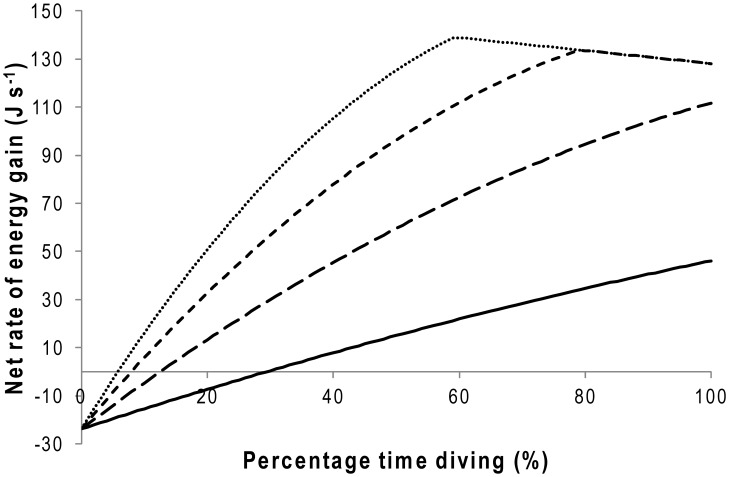
Net rate of energy gain as function of the percentage of time diving. With a digestive physiology defined by Figure 4 and consecrating varying times to foraging underwater in areas with different prey densities. The model assumes that the cost of foraging underwater is 50.7 W, the cost of resting at the sea surface between dives is 23.8 W and that ingestion rate is a linear function of the time spent underwater (500 J s^−1^ - solid line, 1000 J s^−1^ - line with long dashes, 1500 J s^−1^ - line with short dashes and 2000 J s^−1^ - dotted line).

Primary in this must be whether, if birds ate food rapidly, they could process it fast enough to avoid a digestive bottleneck prohibiting further consumption [58]. Research has shown that penguins have an increasing rate of gastric emptying with increasing meal mass (for African penguins) [59] and that increases in consumption also lead to an increase in the rate of faeces production (for Humboldt penguins (*Spheniscus humboldti*)) [5]. Based on this, we can investigate necessary digestion rates implicated by the calculated ingestion rates and their consequences using a simple model. We assume that Magellanic penguins are similar to Humboldt and African penguins [5], [59] in having a throughput rate that is linearly related to the consumption rate (Fig. 4) but that, since this cannot go on indefinitely, this will reach an asymptote that represents the highest throughout rate (Fig. 4). Higher throughput rates result in less time for digestion to take place so that higher throughput rates are associated with lower assimilation efficiencies [60], [61], and we also take this process to be linear (Fig. 4). The rate of energy gain is given by the elimination rate multiplied by the assimilation efficiency, and will always give a logarithmic-type relationship with increasing consumption rates (Fig. 4). The rate of energy gain will also depend on the prey density in the foraging area and the time the birds spend underwater, both of which can be effectively expressed as a rate of energy gain per unit time spent underwater (Fig. 5). If we simplify the costs for activities at sea to 50.7 W for diving and 23.8 W for the cost of resting at the sea surface between dives [45] we can examine how time consecrated to diving in areas with different prey abundance can relate to net energy gain (Fig. 5).

Although not intended to be properly quantitative, not least by virtue of the assumptions, this model shows a number of relevant features; (i) that there is a minimum prey density, below which no amount of diving is energetically beneficial, (ii) that lower prey densities necessitate that birds spend proportionately longer underwater to maximize net gain, (iii) that birds can generally increase their net gain by spending longer periods underwater, (iv) that premise (iii) holds even as prey density increases but that (iv) when prey densities are extremely high, there comes a point when energetic returns decrease with increasing time spent underwater (Fig. 5).

These findings at once explain why deeper-diving birds should generally either have higher wiggle rates during the bottom phase (Fig. 1) but also point to misconceptions relating to how hard penguins work to forage when the rate at which dives are executed is considered. In fact, the frequency of dives executed per unit time is only a useful measure of foraging effort if properly corrected for foraging depth. Deep-diving birds spend longer underwater per dive [e.g. [22],[62]] and longer at the surface recovering from dives [e.g. [22],[63]] so assessment of foraging effort should be cognisant of the decreasing efficiency of penguins foraging at increasing depth (see [22]).

### Colony-specific Prey Consumption

Our derived figures for prey consumption are extreme but if they are correct, how would they translate into colony-specific and area-specific rates of prey removal? The four study colonies have populations estimated at 56737 (Punta Norte), 32337 (Bahía Bustamante), 20287 (Puerto Deseado), and 56792 (Puerto San Julián) breeding pairs [31]. If we consider that during the period of the breeding cycle when our study was undertaken (adults brooding small downy chicks) that one member of the breeding pair goes to sea each day (each pair member would nominally have a day spent brooding the chicks and a day at sea; [36] and references therein) consuming food, the amount consumed per day by each colony would be the number of breeding pairs in this location multiplied by the average amount consumed per day (derived from our data from Table 3 and the mean trip duration by colony according to Sala et al. [46]; see above). Thus, all birds at Punta Norte, Bahía Bustamante, Puerto Deseado and Puerto San Julián colonies, consuming 5.71, 5.24, 3.86 and 3.83 kg day^−1^, respectively, would take 324.0, 169.4, 78.4 and 217.6 tonnes day^−1^, respectively. Birds from these colonies use areas amounting to 2090, 2525, 1188, and 1063 km^2^, respectively [46], and so would be removing something less than about 155.0, 67.1, 66.0, and 204.7 kg km^−2^ day^−1^, respectively. If we assume that the world Magellanic penguin breeding population is 1.3 million pairs [64], and that they consume food at a rate comparable to the calculated mean of our birds of 4.66 kg over one day at-sea, then the whole breeding population would remove over 6000 tonnes of food per day, which, scaled up to the year (ignoring differences that might occur over the course of the year, thus making any calculation very approximate) would be over two million tonnes. Of this amount, over 1.5 millions tonnes (4194 tonnes day^−1^) would be removed by the Argentinian breeding popupation, estimated at 900,000 pairs [31], [64]. Such a harvest would constitute about 87% more than the last ten-year average (i.e. 2000–2010) of total commercial catches per year registered for the main Magellanic penguin prey species (i.e. Argentine anchovy, Argentine hake, Fuegian sprat, Squids, Octopuses, and other marine fishes) in the large area of Southwest Atantic Ocean (*ca.* 820,000 tonnes; [65]).

### Perspectives

The amounts calculated consumed by the Magellanic penguins in this study seem impossibly high so obviously extreme caution must be exercised with the derived data. However, equally, there is increasing evidence that seabirds, at least, consume larger quantities of food than previously estimated [e.g. [56]], suggesting that the premise of equating energy expenditure with energy intake, assuming constant assimilation efficiency, may not be correct either. Our simple model of digestion suggests that ingestion of large quantities of prey by penguins is a strategy that would benefit them energetically although there are diminishing returns. Importantly, it also suggests a mechanism by which penguins may impact prey stocks minimally when prey abundance is low, and much more when prey abundance is high. This feedback system would be density dependent and provide stability in marine ecosystems and is something that could operate on a day to day basis. This is very distinct from fishing policy [e.g. [66],[67],[68]].

Although our approach has involved numerous assumptions, both in terms of prey ingestion rate calculations as well as with regard to rates of digestion, it does highlight possible mechanisms that might explain our outputs, and points to avenues for future research to refute or back-up our suppositions. Given the substantial implications that our calculated rates of penguin food consumption have for ecosystem management, we suggest that it is now critically important for researchers to concentrate effort into methods of determining food consumption by seabirds, especially penguins, by direct methods. There are a number of systems that have been proposed (cf. [8], [17] and references therein), many of which are a few years old and can perhaps now be bettered using newer technology. This would help resolve this extraordinary issue one way or the other.
